# Amino Acid Supplementation to Reduce Environmental Impacts of Broiler and Pig Production: A Review

**DOI:** 10.3389/fvets.2021.689259

**Published:** 2021-07-26

**Authors:** Léa Cappelaere, Josselin Le Cour Grandmaison, Nicolas Martin, William Lambert

**Affiliations:** METEX NOOVISTAGO, Paris, France

**Keywords:** crude protein, amino acids, broiler, pig, nitrogen, environmental impacts, life cycle assessment

## Abstract

Poultry and swine farming are large contributors to environmental impacts, such as climate change, eutrophication, acidification, and air and water pollution. Feed production and manure management are identified as the main sources of these impacts. Reducing dietary crude protein levels is a nutritional strategy recognized to both decrease the use of high-impact feed ingredients and alter manure composition, reducing emissions of harmful components. For a successful implementation of this technique, feed-grade amino acid supplementation is crucial to maintaining animal performance. Reducing crude protein lowers nitrogen excretion, especially excess nitrogen excreted in urea or uric acid form, improving nitrogen efficiency. At the feed-gate, low–crude protein diets can reduce the carbon footprint of feed production through changes in raw material inclusion. The magnitude of this reduction mainly depends on the climate change impact of soybean meal and its land-use change on the feed-grade amino acids used. Reducing dietary crude protein also lowers the environmental impact of manure management in housing, storage, and at spreading: nitrogen emissions from manure (ammonia, nitrates, nitrous oxide) are reduced through reduction of nitrogen excretion. Moreover, synergetic effects exist with nitrogen form, water excretion, and manure pH, further reducing emissions. Volatilization of nitrogen is more reduced in poultry than in pigs, but emissions are more studied and better understood for pig slurry than poultry litter. Ammonia emissions are also more documented than other N-compounds. Low–crude protein diets supplemented with amino acids is a strategy reducing environmental impact at different stages of animal production, making life cycle assessment the best-suited tool to quantify reduction of environmental impacts. Recent studies report an efficient reduction of environmental impacts with low–crude protein diets. However, more standardization of limits and methods used is necessary to compare results. This review summarizes the current knowledge on mitigation of environmental impacts with low–crude protein diets supplemented with amino acids in poultry and swine, its quantification, and the biological mechanisms involved. A comparison between pigs and poultry is also included. It provides concrete information based on quantified research for decision making for the livestock industry and policy makers.

## Introduction

The environmental impact from animal production has become a major concern in the past decades (1, 2). Simultaneously, an increasing world population and shift toward more meat-based diets in developing countries will increase demand for animal products by an estimated 50% by 2050 (3). Pork and chicken are the most consumed meats today and will continue to grow (4), making the transition to less impactful practices crucial for these productions. Furthermore, societal demand for environmentally friendly production is rising and should be taken into account by industry actors in the sector of broiler and pig production.

**Figure 1:**
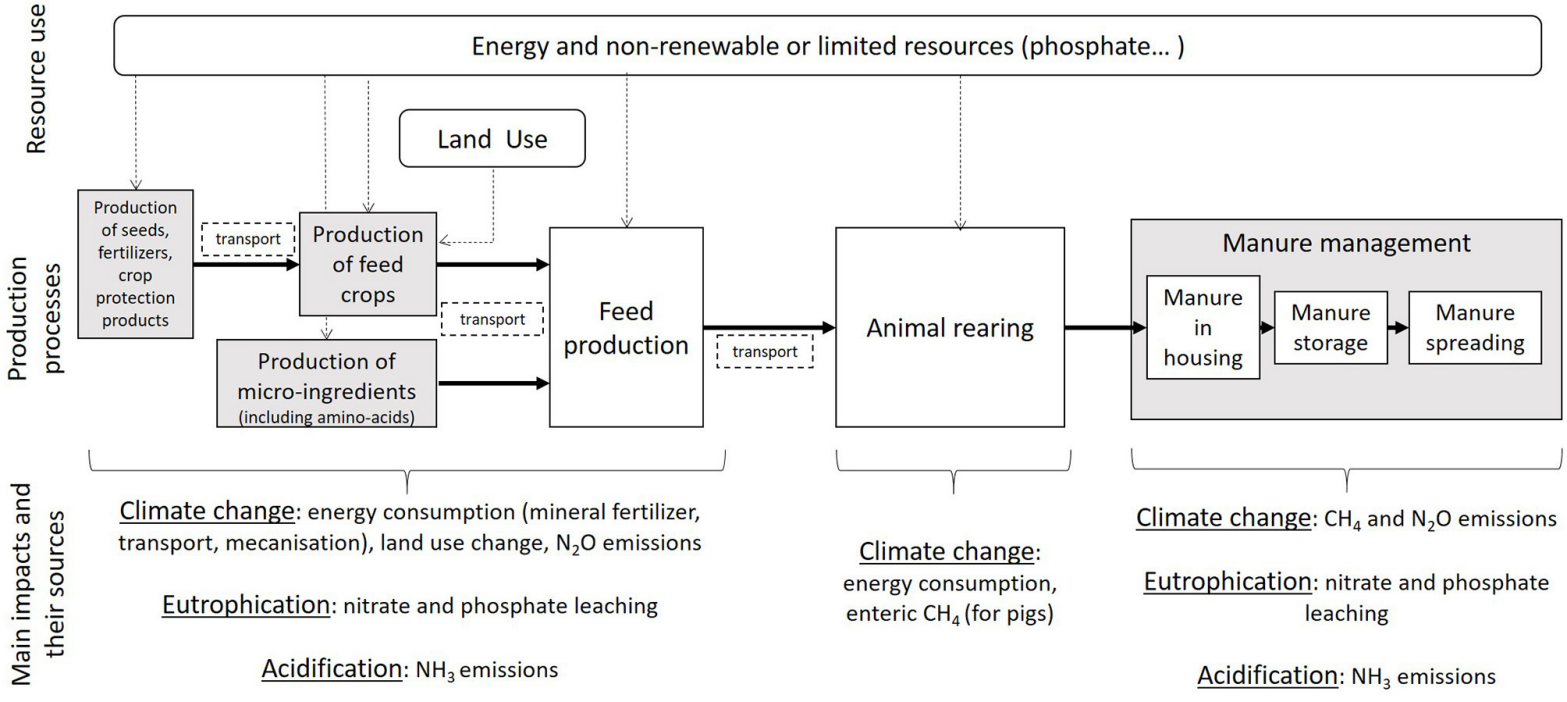
Resource use and environmental impacts associated with the main steps of broiler and pig production.

Environmental impacts of animal rearing are mostly caused by feed production and emissions from manure (5, 6). At the feed production step, the main impacts are climate change linked to energy consumption, nitrous oxide emissions from the fields, and the land-use change (LUC) impact of crops cultivated on recently converted forests or grasslands. This mostly concerns soybean meal (SBM) produced in South America and used widely in Europe and Asia as a source of protein for animal feed. Emissions from the field, due to fertilization, are also an important contributor to acidification and eutrophication. Emissions from manure cause climate change (methane, nitrous oxide), acidification [ammonia (NH_3_)], eutrophication (phosphorus, nitrates, NH_3_), air pollution (NH_3_, particles), and water pollution (nitrates). Nitrogen (N) emissions are involved in all those impacts and are the leading cause of pollution from broiler and pig manure. Those emissions can happen on the farm, in the barn or during manure storage, or at the field after spreading. Other sources of environmental impact are less important in the case of monogastrics. They include energy consumption on the farm and production of enteric methane for pigs. The main processes involved in broiler and pig production and their associated resource use and impacts are summarized in Figure 1.

Nutrition is one of the most effective levers to reduce environmental impact as it can affect emissions from feed production and modify manure composition, limiting emissions in housing and during storage and spreading. Reduction of crude protein (CP) content of feed is a method that has been widely studied and implemented in pig production in order to reduce environmental impacts (7, 8). For broilers, it has been explored more recently with studies focusing on animal performance rather than on implications for environmental impacts, and its practical implementation is still in the early stages (9, 10). In the European Union, it is recognized as a best available technique to reduce NH_3_ emissions from pig and broiler farms (11). It is also a recommended method to reduce eutrophication impact due to nitrate leaching. Indeed, it allows reducing N excretion with a maintained animal performance thanks to feed-grade amino acid (AA) supplementation to cover animal requirements. This reduces N emissions from manure. Low-CP diets supplemented with AA gradually replace protein sources, generally SBM, with cereals and feed-grade AA and possibly alternative protein sources and co-products. In contexts in which SBM associated with LUC is used, this allows tackling the environmental impact of feed production.

This work aims to review the current knowledge on the mitigating effects of low-CP diets supplemented with AA on the environmental impacts of broiler and pig production. Effects of this strategy are considered at different stages of production: animal performance and excretion, feed production, and manure management. Evaluations of the strategy through life cycle assessment (LCA) are also presented, which allows evaluating the effects on the whole system. The review covers both mechanisms involved and available quantification, highlighting areas in which more research is needed and comparing effects between the two species.

## Impact of Low-CP Diets and Feed-Grade AA Inclusion on Animal Performance and Metabolism

### Animal Performance

Dietary CP reduction in swine and broiler diets is performed by replacing protein-rich feedstuffs, generally SBM, by cereals and feed-grade AA. This reduces CP and, thus, N content of the diet while maintaining an adequate supply of indispensable AA.

Low-CP diets formulated with an adequate dietary AA supply are shown to maintain growth performance of growing and finishing pigs consistently in the past decades (12–19). These results are confirmed in recent trials even with a dietary CP reduction of more than 30 g/kg (20–22). Feed-grade AA supplementation is shown to be essential to ensure constant animal performance and successful implementation of a low-CP strategy in swine (22, 23).

In broilers, multiple recently published trials show that reducing dietary CP formulated with an adequate dietary AA supply does not affect growth performance (9, 10, 24–26). When balancing all indispensable AA, it seems possible to reduce dietary CP by 30 g/kg in broiler chickens without affecting growth, intake, feed efficiency, or carcass traits. Lowering dietary CP is also shown to improve animal welfare based on foot pad lesion indicators (10), thanks to a lower litter moisture.

In both species, lowering dietary CP requires a holistic nutritional approach as not only protein and AA, but also fiber, electrolyte balance, and energy sources are affected (27). A careful control of those parameters is, thus, recommended to optimize performance of pigs and poultry fed low-CP diets.

### Nitrogen Balance and Animal Metabolism

**Figure 2:**
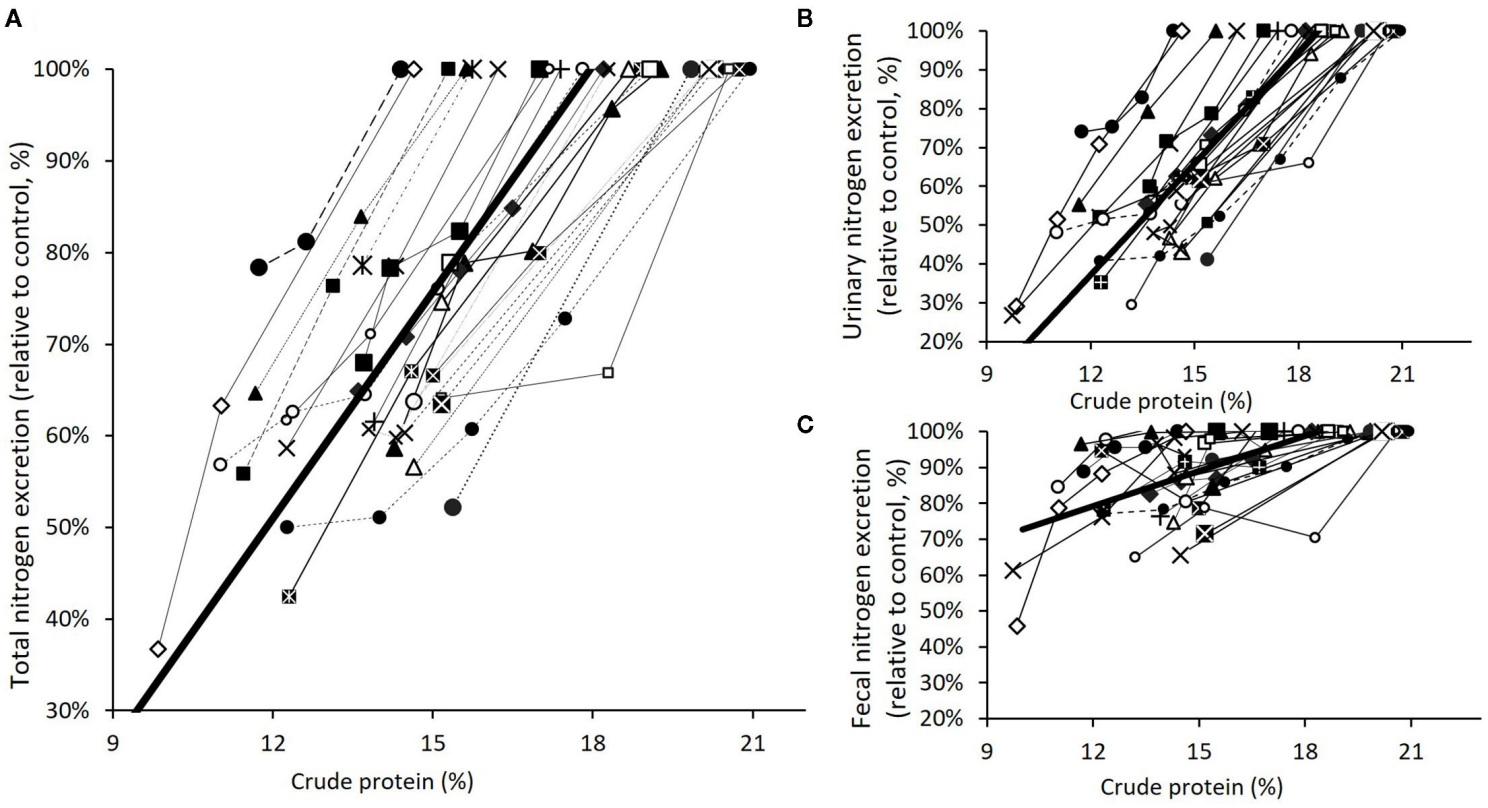
Meta-analysis of the effect of dietary CP reduction on nitrogen excretion in pigs: total excretion **(A)**, urinary excretion **(B)** and fecal excretion **(C)** [adapted from Cappelaere et al. (8)].

**Figure 3:**
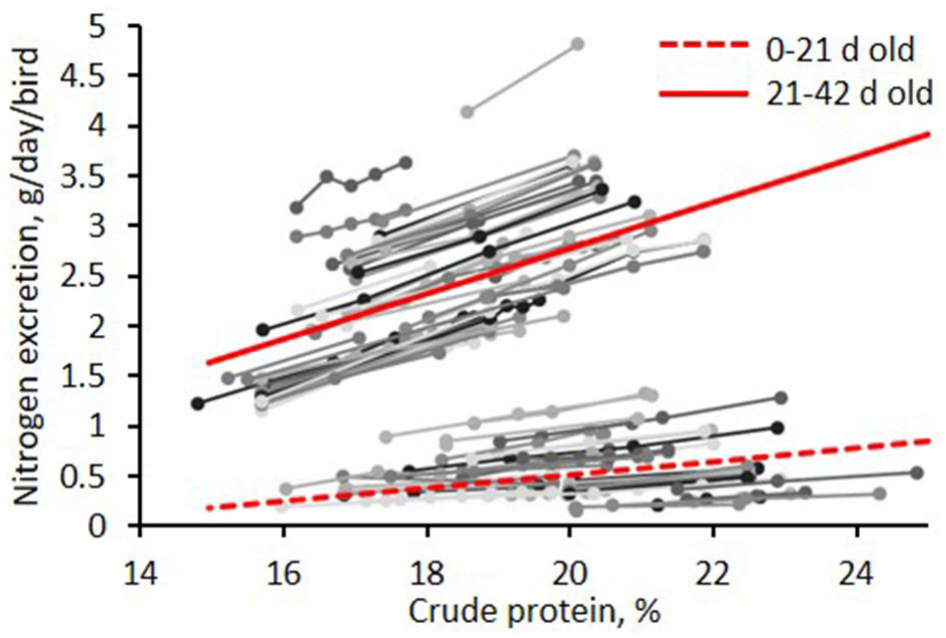
Meta-analysis of the effect of dietary CP reduction on nitrogen excretion in broilers [adapted from Alfonso-Avila et al. (27)].

Various meta-analyses have been published in recent years to synthesize the extensive literature available on the effect of CP reduction on N excretion for both pigs and broilers. In swine, one estimated from nine trials shows a reduction of N excretion by 7.5% per each 10 g/kg CP reduction (28); another reported from 59 publications an average reduction of N excretion of 28.5% with low-CP strategies (29); and the last, based on 27 trials, shows a reduction of N excretion by 8.2% per each 10 g/kg CP reduction (8) (Figure 2). In broilers, a reduction of N excretion by 9% per each 10 g/kg CP reduction was estimated from 107 trials (27) (Figure 3). The reduction of total N excretion with low CP diets is, thus, of similar extent in broilers and pigs.

Reduction of dietary CP lowers N content of diets and, thus, N intake. As growth performance is not affected, N retention is kept constant. Consequently, N efficiency is improved by 1.6 percentage points per each 10 g/kg CP reduction in swine (8) and 2.3 percentage points in broilers (27). Dietary CP reduction with AA supplementation improves valorization of N from feedstuffs used by livestock.

Dietary CP reduction lowers the supply of excess AA, thanks to a better balanced, indispensable AA profile reached with the use of feed-grade AA and a reduction of the dispensable AA content of the diet. This reduces the AA catabolism as evidenced by the reduction of plasma uric acid concentration in broilers (30–33) and serum or plasma urea N concentration in pigs (16, 18, 34). Indeed, N excretion pathways differ between broilers and pigs. Mammals are ureotelic animals, and birds are uricotelic, meaning that N is excreted mainly as urea in the former and as uric acid in the latter. Urea excretion requires more water than uric acid excretion as the first has to be solubilized in urine at a non-toxic concentration while the second is not soluble, less toxic, and excreted directly in solid form in the cloaca, mixed with feces. Conversely, nitrogen excretion in birds is more complex and requires more energy than in mammals.

In pigs, the separation between undigested N excreted in feces and catabolized N excreted in urine allows for easily measuring the contribution of reduction of excess dietary AA to lower N excretion. It also allows differentiating between organic N, which is a stable form of N, and urea N, quickly degraded into ammonia. A meta-analysis (29) reports an average reduction of urinary N excretion by 39.6% with low-CP strategies although fecal N excretion was only reduced by 10.4%. Similarly, another meta-analysis (8) reports a reduction of urinary N excretion by 10% and of fecal N excretion by 3.1% per each 10 g/kg CP reduction (Figure 2). Thus, the share of N excreted as urea is reduced by 2.5 percentage points per each 10 g/kg CP reduction. This meta-analysis also shows that the share of N excreted as urea is very well-predicted by N efficiency, tightly correlated to excess N. A limited effect of classic CP-reduction strategies on fecal excretion is explained by a limited impact on N digestibility. This is not the case when fiber-rich ingredients, such as rapeseed meal, dried distiller's grain with solubles or sugar beet pulp, are used, resulting in a more important shift from urinary to fecal N excretion and an increased fecal excretion (35–37).

This differentiation is not possible in broilers as undigested and catabolized N are excreted together. However, the share of N excreted as uric acid should also decrease in broilers fed low-CP diets as the biological mechanisms involved are similar in broilers and pigs.

Dietary CP reduction also leads to lower water intake and water excretion in both pigs and broilers (27, 38–41). Lower AA catabolism reduces the quantity of water needed for N excretion in both species (42, 43). Dietary CP reduction is also associated with a lower potassium content and electrolyte balance as SBM is a high contributor to dietary potassium, and feed-grade lysine is rich in chlorure. This has the added advantage of lower water intake and excretion with low-CP diets (27, 44, 45).

## Environmental Impact of Feed Production for Low-CP Diets Supplemented With AA

Feed production is the main contributor to the climate change impact of pig and broiler production, accounting for around 60–85% (46–48). These data are consistent across the literature as the evaluation of climate change impacts has been harmonized, following IPCC guidelines (49). Contribution of feed production to the final product's acidification and eutrophication impacts is also significant but varies depending on the allocation of emissions from manure to animal or vegetal production. Furthermore, this contribution reflects the variety of practices and also the characterization methods used for those impacts (50) that are less robust and homogenized between official methods than for climate change (51).

Several feed LCAs focusing on low-CP diets have been performed in recent years for pigs and broilers and are summarized in Table 1. Most of those studies are performed in a European context and test the effect of taking into consideration or not LUC. All studies that included a LUC impact reported a decrease in climate change potential when reducing CP levels, but this decrease was always <5% per each 10 g/kg CP reduction. CP reduction and feed-grade AA inclusion also decrease energy use for feed production (52, 55). The effect on acidification potential is contrasted between publications. It mostly depends on NH_3_ volatilization during crop fertilization and, thus, on agricultural practices considered. Eutrophication potential is consistently decreased with dietary CP reduction.

**Table 1:** Methodology and results of recent broiler and pig feed LCAs.

**publication**	**animals considered**	**farm location**	**crop origin**	**LUC**	**scenario**	**CP levels**	**climate change**	**acidification**	**eutrophication**	**energy demand**
Méda et al. (52)	finishing broilers	France	Europe	yes						
					S19	19	100%	100%	100%	100%
					S17	17	91%	103%	96%	100%
					S15	15	80%	105%	92%	99%
Cherubini et al. (53)	finishing pigs	Brazil	Brazil	no						
						18	100%			
						16	103%			
						15	107%			
						13	110%			
Meul at al. (54)	fattening pigs	Europe	Europe, Brazilian SBM	no						
					reference	15.7	100%			
					low crude protein	13	102%			
				yes						
					reference	15.7	100%			
					low crude protein	13	93%			
				yes + indirect LUC						
					reference	15.7	100%			
					low crude protein	13	91%			
Mosnier et al. (55)	fattening pigs	France	Europe, Brazilian SBM	no						
					standard (noAA)		100%	100%	100%	100%
					biphase (noAA)		99%	98%	98%	94%
					biphase low CP	16.5/15	101%	90%	87%	94%
					biphase least cost with AA		101%	90%	87%	94%
	broilers	France	Europe, Brazilian SBM	no						
					only Met		100%	100%	100%	100%
					Met, Lys		100%	98%	98%	100%
					Met, Lys, Thr		105%	93%	94%	100%
	fattening pigs	France	Europe, Brazilian SBM	yes						
					standard (noAA)		100%	100%	100%	100%
					biphase (noAA)		97%	96%	98%	94%
					biphase low CP	16.5/15	94%	87%	89%	88%
					biphase least cost with AA		93%	85%	89%	86%
	broilers	France	Europe, Brazilian SBM	yes						
					only Met		100%	100%	100%	100%
					Met, Lys		98%	97%	98%	97%
					Met, Lys, Thr		100%	92%	94%	96%

Dietary CP reduction generally replaces SBM with cereals and feed-grade AA. SBM is mostly produced in North and South America. South American SBM is generally associated with deforestation and has a high LUC impact, especially in Center-West Brazil, leading to a high climate change impact (56, 57). This is not the case for SBM produced in North America. Thus, dietary CP reduction is mainly implemented to reduce the climate change impact of feed when Brazilian SBM is used.

**Figure 4:**
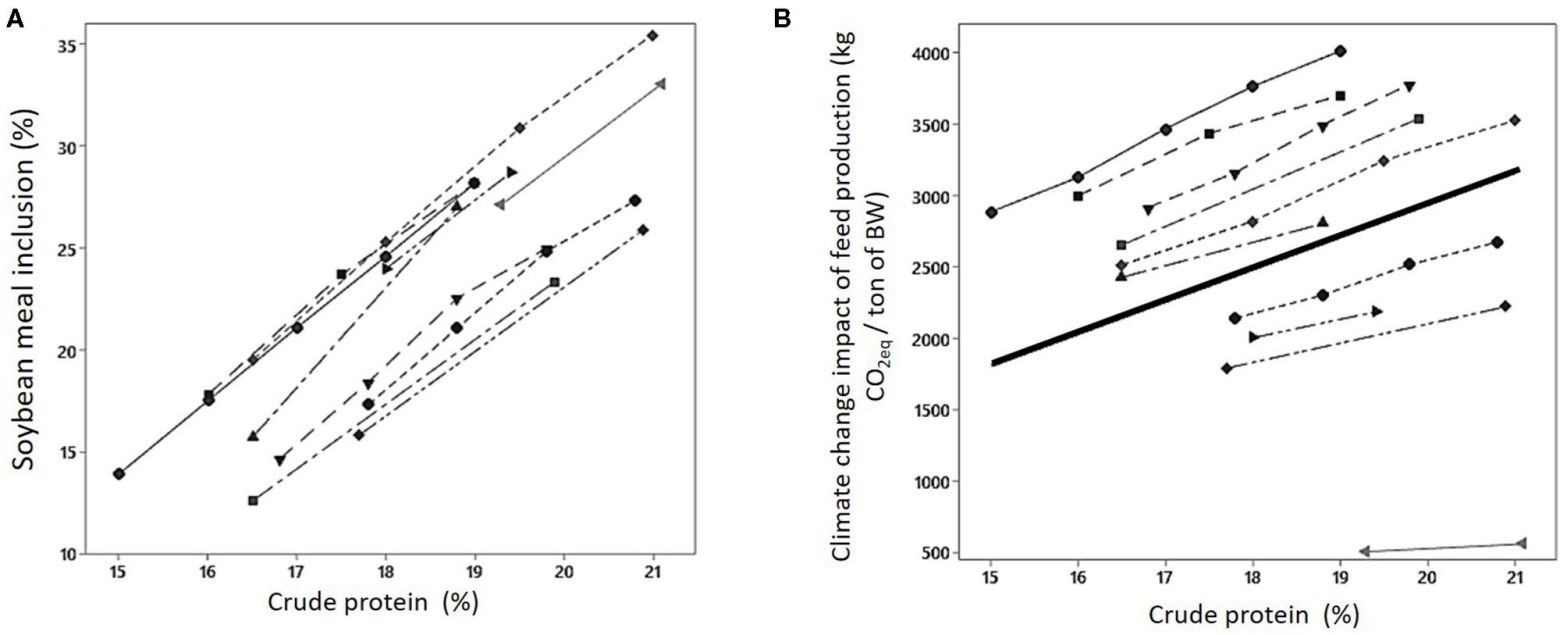
Effect of CP reduction and supplementation with AA on SBM use **(A)** and climate change impact of feed production **(B)** [adapted from Le Cour Grandmaison et al. (61, 62)].

In contexts in which the SBM used has an LUC impact (South America, Europe, China), CP reduction allows reducing the climate change impact associated with SBM inclusion. In low-CP diets, impact values attributed to feed-grade AA are also important even if their inclusion rate is low because they are much higher compared with crops due to the processes used and the high energy demand (55, 58). These values can vary with the origin of the product, type of energy used, and C and N sources for fermentation. Due to these influencing factors, the climate change impact of feed production has been shown to slightly increase with CP reduction in some specific contexts (53, 59). Monteiro et al. (60) shows that, with a farm-gate LCA, CP reduction and AA inclusion decreased climate change impact of French and Brazilian pig production when SBM associated with recent deforestation was used but increased it when SBM was not associated with LUC. Furthermore, the effect of the strategy was more pronounced when SBM was the sole source of protein compared with diets with a mix of protein sources. Similarly, Kebreab et al. (48) evaluate the sensitivity to geographical context and inclusion or not of LUC of the benefits from AA inclusion in pig and broiler production. When LUC was excluded, variation of the feed production climate change impact was low and depended on the geographical region and the species, whereas when LUC was included, climate change impact consistently decreased. In a European context using Brazilian SBM associated with LUC, Le Cour Grandmaison et al. (61) calculated, from recent performance trials, that a 10 g/kg dietary CP reduction reduced climate change impact of a ton of feed by 101 kg CO_2_eq. It corresponds to an 8% decrease of climate change impact and, for broilers, to a reduction of 226 kg CO_2_eq per ton of live weight (62). This is associated with a reduction of SBM inclusion of 39 kg/t in fattening pigs and 35 kg/t in broilers for each point of dietary CP reduction (Figure 4).

Beyond the context of production, quantitative benefits of low-CP strategies for the climate change impact of diets relies heavily on the value taken for LUC impact of SBM. Those values vary greatly between LCA databases, even for equivalent products. For example, the average Brazilian SBM climate change impact triples between EcoAlim (ADEME, 2016) and GFLI (Global Feed LCA institute, 2019) or Agri-footprint 5.0 (Blonk Consultants, 2019) databases. Da Silva et al. (57) also frequently uses in LCAs reports of even lower climate change impacts with a low contribution of LUC (under 30%). Climate change impact values of Brazilian soybeans are presented in Table 2. Efforts have been made to propose a standardized method to calculate LUC (63), and those guidelines should be broadly used to improve the consistency and comparability of studies.

**Table 2:** Average Brazilian soybean climate change value in several databases and publications.

	**AgriFootPrint 5.0 and GFLI**	**EcoInvent 3.6**	**EcoAlim V7**	**Da Silva et al. (57)**
Climate change impact (kg CO_2_eq/kg)	5.6	2.6	1.35	0.51–0.96

Some approaches have also taken into consideration indirect LUC, i.e., LUC caused by the displacement of other crops, pushed by the increased demand for crop cultivated on existing cropland (54), reducing the benefits of using feedstuffs from non-deforested areas. Beyond LUC, CP reduction is shown to reduce land use by up to 10% (64, 65), reducing the pressure for arable land in all production regions.

Reducing CP level using AA also allows for more flexibility on raw materials used. Thus, inclusion of alternative protein sources is made easier, permitting an increase in use of local feedstuffs and reducing the impact of feed transportation and also to valorize more by-products (54).

## Low-CP Strategies Reduce Environmental Impact of Manure Management

### Composition of Manure

Commercial broilers across the world are mainly reared on litter, and manure is managed in solid form (66). Conversely, most pigs are reared on slatted floors, and urine and feces are mixed to produce liquid manure, i.e., slurry (11). Feeding animals low-CP diets reduces their N excretion and, in turn, manure N content. A reduction of total N content of pig manure by 3.5% per each 10 g/kg CP reduction was quantified (67). A meta-analysis calculated a higher reduction of N concentration of pig slurry by 5% per each 10 g/kg CP reduction (8), which could be explained by the inclusion of more recent experiments and the selection of trials on iso-digestible lysine diets. Litter N content has also been shown to decrease with low-CP diets in broilers (9, 10, 33, 68).

The chemical form of N in manure is affected by CP reduction. Uric acid and urea, to a greater extent, are quickly degraded by bacteria into NH_3_, and N contained in undigested proteins is very stable. Consequently, total ammoniacal N (TAN) content of manure is greatly reduced with low-CP diets correctly supplemented with feed-grade AA. Meta-analyses have estimated a reduction of TAN content of pig slurry by 7–8% per each 10 g/kg CP reduction (8, 69). In broilers, litter TAN has been less studied, and more research is needed on this parameter. An article reported no statistical effect of CP reduction on litter TAN content (10) although a trial in breeders showed a 9% reduction of TAN concentration with a 15 g/kg CP reduction (70).

The lower water excretion of animals with low-CP diets reduces manure moisture and increases dry matter (DM) content and, consequently, reduces manure production expressed as volume or weight. In pigs, slurry volume is reduced by 2.8% per each 10 g/kg CP reduction (8). Some studies report a higher DM content of slurry with low-CP diets (39, 71), but other dietary parameters, such as fiber content, have a higher impact and interact with the protein level (72, 73). Hence, the impact on DM content depends on the formulation choices made when implementing CP reduction and their consequences on ingredient inclusion. In broilers, a reduction of litter moisture by 12 g/kg and a reduction of litter weight by 3.3% per each 10 g/kg CP reduction was quantified by meta-analysis (27). Reduction of CP results in drier and more friable litter with a higher DM content (10).

The magnitude of the benefits, during manure management, of changes in manure composition due to nutritional strategies depends on manure management practices implemented (slurry separation, composting, biological treatment, etc.).

Modifications of pig slurry composition with manipulation of dietary CP have been modeled (74), allowing a precise prediction of the effects of nutritional strategies. Such an approach has not been yet developed in broilers.

Dietary CP reduction also reduces slurry pH in swine (67) by 0.15 points per each 10 g/kg CP reduction (8). This can be explained by a lowered urinary pH (38, 75) due to a lower electrolytic balance. Fecal pH can also be lowered when alternative protein sources rich in fiber are added as hindgut fermentation producing volatile fatty acids is increased (36, 76). In broilers, no effect of CP level on litter pH has been found (10, 68).

### Volatilization of N Components and Biological Processes Involved

**Figure 5:**
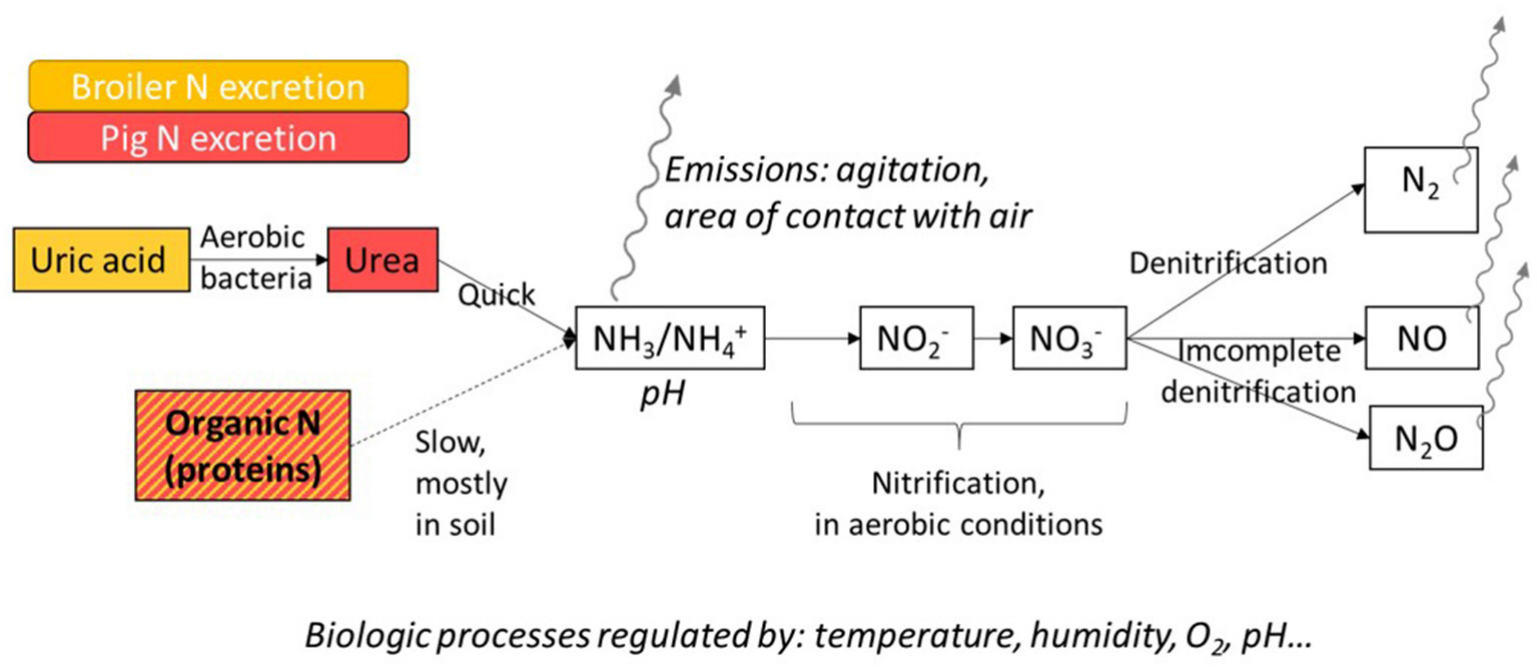
Biochemical processes of the degradation of nitrogen excreted by pigs and broilers.

The nitrogen content of pig and broiler manure is degraded by microorganisms, resulting in different N compounds emitted in the environment with various negative impacts. When manure is produced, the main forms of N are undigested and microbial protein (organic N), urea or uric acid, depending on the species (simple forms of organic N), and ammoniacal N (mineral N). Uric acid is degraded into urea by aerobic bacteria. Urea is quickly degraded into NH_3_ by urease produced by microorganisms present in manure. Mineralization of undigested protein is slower and requires specific organisms. It happens mostly in soils when manure storage is short. With long-term storage of slurry or litter or when it undergoes treatments, such as composting, a significant share of the organic matter is degraded before spreading. Ammoniacal N is present in manure in an acid-base equilibrium between ammonium (${\text{NH}}_{4}^{+}$) and NH_3_. It is degraded into N oxides: nitrites (${\text{NO}}_{2}^{-}$) and then nitrates (${\text{NO}}_{3}^{-}$) during the nitrification process. This step of the N cycle takes place in aerobic conditions and, thus, mainly concerns litter compared with slurry, which is an anaerobic environment. Nitrates are degraded into N_2_ during denitrification. Nitric oxide (NO) and nitrous oxide (N_2_O) are also produced during this nitrification–denitrification process due to incomplete biological reactions. Intensity of these processes depends on numerous regulating factors of microbial activity: substrate availability, aerobic or anaerobic conditions, humidity, temperature, pH. Gaseous N compounds (NH_3_, NO, N_2_O, N_2_) are volatilized when exposed to the atmosphere. As slurry is an anaerobic environment, on-farm emissions are in NH_3_ form for the majority. In poultry litter, the entire N mineralization process can take place, and emissions are more diversified. The volatilization rate depends on chemical and physical parameters, such as temperature, pH, concentration, and air flow (77). These mechanisms are summed up in Figure 5.

The first effect of CP reduction is to decrease manure N content and, thus, substrate to produce harmful N compounds, particularly TAN, as presented above. Synergies with other impacts on manure composition are also involved as CP reduction influences humidity and pH of manure as well as C/N ratio. In pigs, the main effect is the one of pH as a more acidic slurry favors the left-hand side of the ${\text{NH}}_{4}^{+}$/NH_3_ chemical equilibrium and limits volatilization of NH_3_. This effect is limited and was not caught by the meta-analysis by (8), as CP reduction was not shown to have an effect on the emission factor of TAN into NH_3_. Water content of slurry does not have a significant effect on ammonia emissions as slurry remains an anaerobic environment in which water is not limiting to biological processes when water content decreases. However, a higher DM content of slurry is shown to reduce emission factors (77). Conversely, water content of broiler litter largely impacts N volatilization (78) as it is a limiting factor for the biological breakdown of uric acid into NH_3_. Belloir et al. (9) measured a reduction of the share of N volatilized from broiler litter with CP reduction of between 3.9 and 6.4 percentage points per each 10 g/kg CP reduction. However, the form in which N was volatilized was not studied, and thus, no conclusion on NH_3_, NO, or N_2_O volatilization can be drawn without strong hypotheses. The effect of litter humidity and pH on emissions introduces a synergetic effect between CP reduction and SBM inclusion reduction as the later reduces dietary potassium and, thus, water intake and excretion as well as electrolytic balance and, thus, excreta pH as detailed in previous parts.

These synergetic effects mostly concern on-farm emissions. After spreading, manure is incorporated into the soil and becomes part of a more complex system with many interfering factors (soil type, climate, meteorological conditions, agronomic practices, etc.).

For pig production, the most studied on-farm emissions are NH_3_ emissions. Meta-analyses show a reduction of on-farm NH_3_ emissions by 10% (8) or 11% (69) per each 10 g/kg CP reduction. Reduction of NH_3_ emissions in the barn and at storage ranging from 24 to 65% with low CP strategies are reported (29, 67).

In poultry, quantification of NH_3_ emissions in the context of low-CP strategies is not as advanced. Reduction of NH_3_ emission by 16% was observed in commercial settings with a 15 g/kg CP reduction (29). A synergy was observed with litter moisture as N excretion was only reduced by 4.8% due to impaired performance. With a similar CP reduction, another trial measured a 9% reduction of NH_3_ emissions and an 11% reduction of total N volatilization with breeder broilers (70). A meta-analysis estimated a reduction of NH_3_ emissions by 20% with low-CP strategies (66).

For other N compounds, empirical data on the effect of CP reduction is scarcer. A reduction of N_2_O emissions from pig manure compost by 39% is shown with a 25 g/kg CP reduction (79) with no effect on the emission factor. Conversely, two studies identify no effect on N_2_O emissions with up to a 30 g/kg CP reduction (80, 81).

The effect of low-CP diets on emissions during and after manure spreading are also rarely studied. Portejoie et al. (39) did so, measuring a 53% reduction of NH_3_ emissions following pig slurry application for a CP reduction from 200 to 120 g/kg but no significant difference between 160 and 200 g/kg CP treatments. Furthermore, models proposed by international guidelines use fixed emission factors—depending on the animal species, type of manure, country, and climate—based on TAN (82) or total N (49). With these hypotheses, reduction of N_2_O, NO, and nitrate emissions are of the same magnitude as reduction of N excretion. However, predicting the fate of N in organic fertilizer is much more complicated as it interacts with the soil and crops. Reducing dietary CP decreases TAN content of manure that is readily available for fertilized crops, and increases the share of organic N, slowly degraded and made available for plants. Without an effective prediction of nutrients available long-term, associated with good fertilization management, changes in manure composition achieved with low-CP strategies can lead to a reduction of emissions at spreading but an increase of long-term emissions, such as nitrate leaching (83).

More research is needed to accurately model the effects of low-CP diets on N emission factors and the type of N compound volatilized. This is particularly true for poultry production and for emissions other than NH_3_.

Associated with the reduction of these emissions, the environmental impact of manure management can be greatly decreased. An LCA study estimated a reduction of the eutrophication potential linked to manure management by 20–35% in pigs and 19–49% in broilers with low-CP diets supplemented with feed-grade AA, depending on the continent considered (48). Acidification potential was reduced by 30–35% in pigs and 51–53% in broilers.

## Farmgate Life Cycle Assessment Allows Aggregating the Value Created Along the Production Chain

Dietary CP reduction is a multifactorial strategy that reduces environmental impacts from feed production and also from manure management. To correctly take into consideration those benefits, the whole pig or broiler production chain has to be assessed, from production of feedstuffs to manure spreading. LCA is the best-suited method to do so, thanks to its normalized methodological framework (84, 85) designed to measure the environmental impact of a product or a system throughout its life cycle with a holistic approach.

Dietary CP reduction strategies have been thoroughly evaluated with LCA approaches in pigs since the early 2000s (86, 87), but they have only been recently studied in broilers with few publications. Supplementary Table 1 summarizes methodology used and results of recent pig LCA studies and available broiler LCA studies focusing on the effect of CP reduction and feed-grade AA supplementation. Similar results were obtained for pigs and broilers. All but one (52) study took into consideration the impact of manure spreading, using the system extension method, meaning that the manure is considered to be used to fertilize crops fed to the animals studied. This allows fully grasping the effect of the mitigation measure. With this method, use of animal manure for fertilization is generally considered to avoid production of mineral fertilizer. This gives a bonus for manure production on energy demand, which is decreased with reduction of N excretion, penalizing low-CP diets for this indicator.

Most studied contexts are European and Brazilian animal productions, which are major broiler and pig producers, but more research effort should be carried out for North American and Asian contexts. The method used was fairly similar between publications. The functional unit used is generally a ton of live weight, but some studies also used the animal (59).

**Figure 6:**
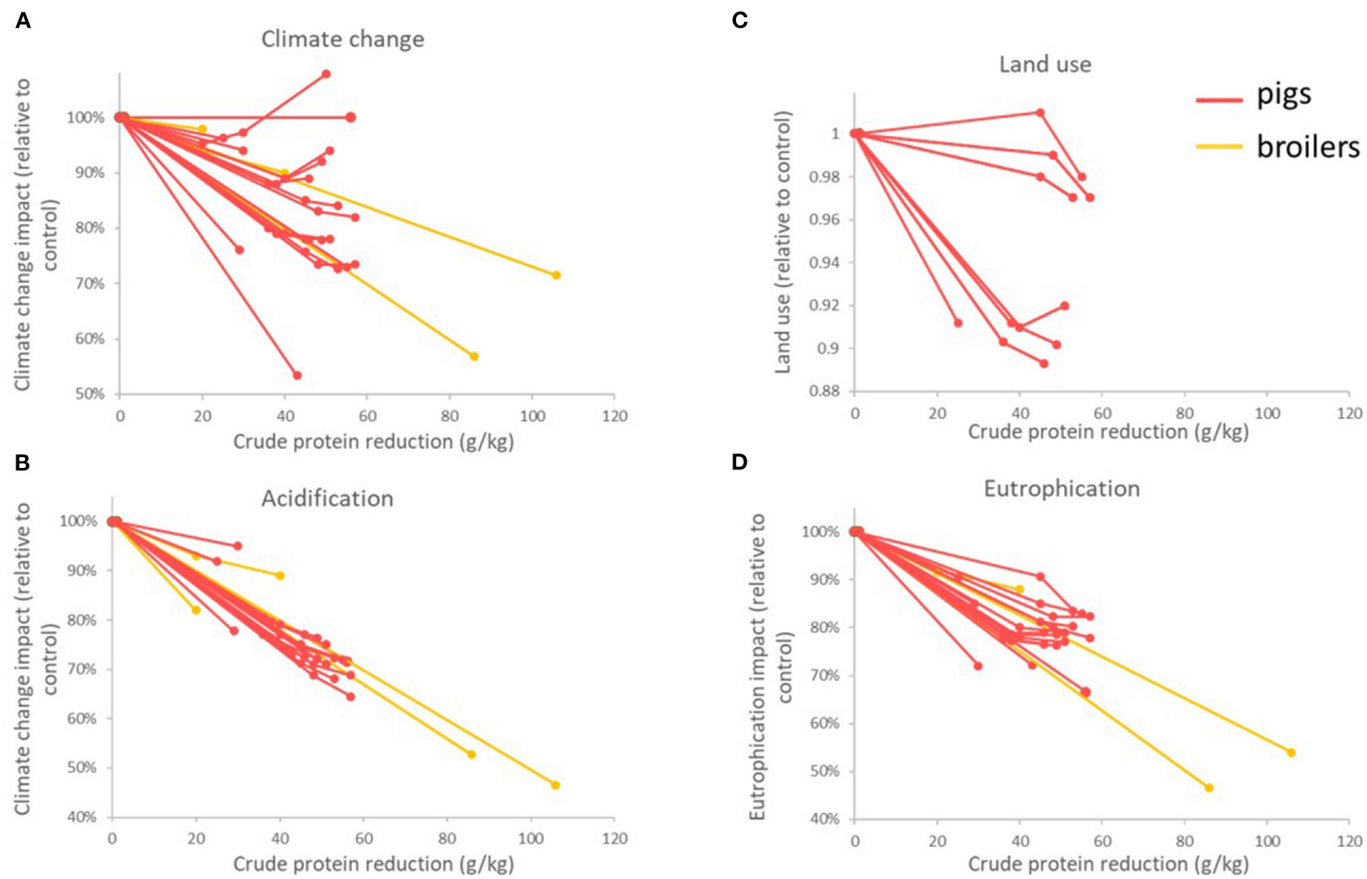
Reduction of environmental impacts with CP reduction in recent pig and broiler LCAs: climate change **(A)**, acidification **(B)**, land use **(C)** and eutrophication **(D)**.

All LCAs considered in this review report a positive effect of CP reduction on the environmental parameters studied (Figure 6): climate change, acidification, eutrophication, and land use. The effect on climate change was low when no LUC was considered (48, 64), confirming the conclusions of feed LCAs. Acidification and eutrophication impacts are consistently reduced (7) regardless of context. This is explained by the fact that those impacts are mainly caused by emissions from manure (48, 65). Energy demand increased in some studies, particularly due to energy used for feed-grade AA production (48, 52, 64). Compatibilization of manure used as fertilizer is also involved.

An LCA (65) studied in a French context showed the effect of numerous interacting factors on reduction of environmental impact with low-CP diets supplemented with feed-grade AA: type of manure management, source of protein, interaction with phase-feeding, and LUC value of SBM. CP reduction had a positive effect in all of the contexts. The main differences between liquid and solid manure management were the contribution of manure emissions to climate change impact with higher emissions of N_2_O from manure in solid manure systems. As a consequence, reduction of climate change impact is more important in solid manure systems. Other factors had a low influence on the effect of nutritional modifications. Climate change impact was highly affected by the origin of SBM and N_2_O emission factors.

## Discussion

### State-of-the-Art and Future Research

Dietary CP reduction is possible without decreasing performance with adequate feed-grade AA supplementation. Very low-CP diets need to be further explored to keep lowering the environmental impact of animal production. Not impairing performance is of the first importance to keep the economic value of animal production and also to improve environmental performance as feed efficiency is one of the first levers to do so (88). Variability in animal performance has been identified to affect results of dietary solutions (60).

The impact of CP reduction on N balance has been largely studied for both species. Accurate quantification of the amount of N excreted with low-CP diets supplemented with feed-grade AA is available and should be used to predict N emissions from manure. However, the form of in which N is found in manure is also important. Reactive N responsible for emissions is mainly in ammoniacal form. In pigs, the share of N in ammoniacal form can be predicted from urinary excretion, and models exist to estimate it with CP reduction. Due to metabolic differences, this is not the case for broilers for which the share of N excreted as uric acid is not well-documented. This discrepancy remains at the manure level as effects of CP reduction on pig slurry composition are well-quantified, and effects on poultry litter remain to be explored. Furthermore, slurry is a simpler environment to study compared with dry manure as only anaerobic processes happen in the former. Several models for N volatilization have been developed for pigs, including an effect of dietary protein or manure N. This does not exist in broilers, and the effect of reducing CP on N emissions has only been quantified in a few trials. Most publications study NH_3_ emissions, but more work is needed for other types of emissions, especially nitrous oxide. Moreover, studies on reducing CP diets focus on emissions at the farm, and few data is available on the effects at the field after spreading. Agronomic valorization of the organic fertilizer produced should also be taken into account to evaluate nutritional strategies. Reducing dietary CP will be more interesting in a territory producing excess organic N fertilizer compared with a territory where it is in demand.

LCA studies on low-CP diets were first performed at the feed level (86). This measures the effects of the strategy on the impact of feed production. The context of production influences greatly the results of all those studies as LUC impact has the highest weight (60). To have comparable data, a standardized method should be used, and LCA values of crops and feed ingredients (especially feed-grade AA and SBM) should be harmonized between databases to avoid cherry-picking of values. The PEFCR method for evaluation of feed provides guidelines and should be used more broadly. In European and South American contexts, a low-CP diet supplemented with amino acids is shown to reduce the environmental impacts of feed production, but the magnitude of this impact is low. In contexts in which SBM associated with recent LUC is not used, low-CP strategies do not intend to reduce environmental impacts of feed production and have a low potential of doing so. In those cases, the interest of low-CP strategies is mostly in the reduction on environmental impacts arising from N excretion.

Thus, it is crucial to aggregate the different effects of the strategy to evaluate its benefits. To fully encompass the effects of low-CP diets on environmental impacts, a farm-scale LCA is the most appropriate tool. It should include the impact of manure spreading to represent accurately the benefits of low-CP diets. However, this is complicated as it requires modifying feed production data to avoid accounting twice for emissions from fertilization. A first step is to, at least, take into account emissions up to manure storage in product LCAs. The LCA method has been increasingly used for pig production since the early 2000s (89). In broiler production, environmental impact has been more recently raised as a concern, and thus, few LCAs are available, even fewer on a specific topic such as low-CP diets supplemented with feed-grade AA (90). The scope and method applied are similar between studies, allowing for comparison. Results are all in accordance to indicate a positive effect of CP reduction on climate change, acidification, and eutrophication impacts.

### Perspectives for the Broiler and Pig Sectors

Dietary CP reduction is an effective method to reduce the environmental impact of broiler and pig production. Protein inclusion has already been substantially decreased in pig diets, especially in European production. This process is only starting for broiler production across the world. Current knowledge on animal nutrition and experimental data leaves room to keep reducing dietary CP levels in both species and, doing so, reducing environmental impact. Innovation, such as precision feeding, will allow further decreasing CP levels as individual requirements will be met. Precision feeding can significantly contribute to improving environmental performance of livestock (91, 92). Synergy with the reduction of dietary CP further drives down the environmental impacts of poultry and pigs. Precision feeding also has the added advantage to be able target multiple problematics, such as phosphorus or energy supply, in a holistic approach.

To keep improving the environmental performance of animal production, quantified impacts should be included in the decision-making process at the farm or on the company scale (93) and for policy making (94). In this context, specificity and accuracy of data used to model animal performance and emissions is key to have reliable quantification of the effects of a strategy (95), and further research is encouraged to fill the gaps identified. Interaction with types of manure management and other mitigating measures has to be studied (precision feeding, technological measures, manure treatment, anaerobic digestion for energy production, etc.) as these techniques have to be combined to achieve optimized environmental performance. To be able to compare mitigating techniques, the same scope should be used for LCAs estimating their benefits (89). The whole animal production system from feed production to manure management should be considered to be as inclusive as possible.

Including feed LCA values in feed formulation is shown to significantly decrease environmental impacts of feed compared with the least-cost formulation currently in use (46, 94). It supports further reduction of CP levels and inclusion of feed-grade AA. To increase accuracy of the predicted impact of dietary solutions on environmental impacts, a farm-scale LCA model should be used, but it is harder to implement as we exit the linear programming domain. Inclusion of other indicators should be also considered to encompass societal and environmental demands: economics, animal health and welfare, and antibiotic use (96).

## Conclusion

Low-CP diets supplemented with feed-grade AA reduce the environmental impact of broiler and pig production, acting on both impact of feed production and emissions from manure. Concerning impact of feed ingredients, the amplitude of the mitigation effect of the strategy depends on the raw material and geographical context. Implementation of harmonized methods is necessary to have reproducible and comparable data. For effects on manure emissions, mechanisms involved and quantification of the effects of CP reduction have been thoroughly studied for pigs. It is not the case for broilers for which only the effect on N excretion is well-quantified. For both species, a higher focus on molecules other than NH_3_ is also needed, especially N_2_O, for which climate change impact is well-known but for which emissions factors have a lot of uncertainty.

## Author Contributions

LC, WL, and NM worked on the conception of the review. LC performed the literature review and drafted the article. WL, JL, and NM provided critical review of the paper. LC, JL, NM, and WL approved the final draft of the paper. All authors contributed to the article and approved the submitted version.

## Conflict of Interest

The authors declare that the research was conducted in the absence of any commercial or financial relationships that could be construed as a potential conflict of interest. This paper was funded and produced by employees of Metex Noovistago, which produces and sells feed-grade amino acids.

## Publisher's Note

All claims expressed in this article are solely those of the authors and do not necessarily represent those of their affiliated organizations, or those of the publisher, the editors and the reviewers. Any product that may be evaluated in this article, or claim that may be made by its manufacturer, is not guaranteed or endorsed by the publisher.
