# Supplementary material for: Amino Acid Supplementation to Reduce Environmental Impacts of Broiler and Pig Production: A Review
Source: Front Vet Sci. 2021 Jul 26;8:689259. doi: 10.3389/fvets.2021.689259 (PMC8350159; doi:10.3389/fvets.2021.689259)
Supplement: Supplementary file 1 [file Table_1.docx]

Table S1 :methodology and results of recent broiler and pig crude protein reduction cradle to farm-gate LCAs

| **publication** | **animals considered** | **functional unit** | **farm location** | **crops origin** | **LUC** | **scenarii** | **CP levels** | **climate change** | **acidification** | **eutrophication** | **energy demand** |
| --- | --- | --- | --- | --- | --- | --- | --- | --- | --- | --- | --- |
| *Méda et al., 2017* | *broilers* | *t LW* | *France* | *Europe* | *yes* | S19 | Control / 19 | 100% | 100% | 100% | 100% |
|  |  |  |  |  |  | S17 | Control / 17 | 94% | 93% | 92% | 100% |
|  |  |  |  |  |  | S15 | Control / 15 | 90% | 89% | 88% | 101% |
| *Monteiro et al., 2017* | *fattening pigs* | *kg BWG* | *Brasil* | *South brasil* | *no* | Standard18.2 / 17.1 / 16.1 / 13.5 | | 100% | 100% | 100% | 100% |
|  |  |  |  |  |  | low nutrient14.8 / 14.6 / 14.4 / 12.5 | | 96% | 92% | 91% | 101% |
| *Kebreab et al., 2016* | *sows + fattening pigs* | *t LW* | *North America* | *North america* | *no* | standard | 21.2 | 100% | 100% | 100% | 100% |
|  |  |  |  |  |  | standard +AA | 15.6 | 100% | 72% | 67% | 112% |
|  |  |  | *South America* | *South america* | *no* | standard | 18.7 | 100% | 100% | 100% | 100% |
|  |  |  |  |  |  | standard +AA | 15.8 | 100% | 72% | 67% | 112% |
|  |  |  | *Europe* | *Europe, brazilian SBM* | *no* | standard | 18.1 | 100% | 100% | 100% | 100% |
|  |  |  |  |  |  | standard +AA | 13.8 | 100% | 73% | 72% | 112% |
|  |  |  | *South America* | *South america* | *yes* | standard | 18.7 | 100% | 100% | 100% | 100% |
|  |  |  |  |  |  | standard +AA | 15.8 | 76% | 78% | 85% | 105% |
|  |  |  | *Europe* | *Europe,brazilian SBM* | *yes* | standard | 18.1 | 100% | 100% | 100% | 100% |
|  |  |  |  |  |  | standard +AA | 13.8 | 53% | 73% | 72% | 112% |
|  | *broilers* | *t LW* | *North America* | *North america* | *no* | standard | 21.9 | 100% | 100% | 100% | 100% |
|  |  |  |  |  |  | standard +AA | 19.9 | 98% | 82% | 92% | 106% |
|  |  |  | *South America* | *South america* | *no* | standard | 29.3 | 100% | 100% | 100% | 100% |
|  |  |  |  |  |  | standard +AA | 18.7 | 94% | 47% | 54% | 73% |
|  |  |  | *Europe* | *Europe,brazilian SBM* | *no* | standard | 26.5 | 100% | 100% | 100% | 100% |
|  |  |  |  |  |  | standard +AA | 17.9 | 88% | 53% | 46% | 90% |
|  |  |  | *South America* | *South america* | *yes* | standard | 29.3 | 100% | 100% | 100% | 100% |
|  |  |  |  |  |  | standard +AA | 18.7 | 71% | 47% | 54% | 73% |
|  |  |  | *Europe* | *Europe,brazilian SBM* | *yes* | standard | 26.5 | 100% | 100% | 100% | 100% |
|  |  |  |  |  |  | standard +AA | 17.9 | 57% | 53% | 46% | 90% |
| *Cherubini et al., 2015* | *finishing pigs* | *30kg gain* | *Brazil* | *Brazil* | *no* |  | 18 | 100% |  |  |  |
|  |  |  |  |  |  |  | 16 | 95% |  |  |  |
|  |  |  |  |  |  |  | 15 | 97% |  |  |  |
|  |  |  |  |  |  |  | 13 | 108% |  |  |  |
| *Garcia-Launay et al., 2014* | *sows + fattening pigs* |  | *France* | *Europe, brazilian SBM* | *yes* |  |  |  |  |  |  |
|  |  |  |  |  | Soybean meal | one phase - no aa | 19.4 | 100% | 100% | 100% | 100% |
|  |  |  |  |  |  | one phase - least cost with aa | 14.9 | 88% | 73% | 82% | 97% |
|  |  |  |  |  |  | one phase - low CP | 16.5 | 92% | 83% | 89% | 97% |
|  |  |  |  |  |  | one phase - minimum CP | 13.9 | 86% | 69% | 80% | 99% |
|  |  |  |  |  |  | biphase - least cost with aa | 13.9 | 85% | 67% | 78% | 94% |
|  |  |  |  |  |  | biphase - low CP | 15.9 | 91% | 79% | 86% | 95% |
|  |  |  |  |  |  | biphase - minimum CP | 13.1 | 84% | 64% | 77% | 96% |
|  |  |  |  |  |  | multiphase - no aa | 18 | 97% | 93% | 95% | 97% |
|  |  |  |  |  |  | multiphase - least cost with aa | 13.2 | 83% | 64% | 76% | 91% |
|  |  |  |  |  |  | multiphase - minimum CP | 12.3 | 82% | 60% | 74% | 93% |
|  |  |  |  |  | Soybean meal + rapeseed meal | one phase - no aa | 19 | 100% | 100% | 100% | 100% |
|  |  |  |  |  |  | one phase - least cost with aa | 15 | 89% | 77% | 78% | 96% |
|  |  |  |  |  |  | one phase - low CP | 16.5 | 93% | 86% | 84% | 97% |
|  |  |  |  |  |  | one phase - minimum CP | 13.9 | 94% | 71% | 77% | 105% |
|  |  |  |  |  |  | biphase - no aa | 18 | 97% | 93% | 97% | 97% |
|  |  |  |  |  |  | biphase - least cost with aa | 14.2 | 88% | 71% | 75% | 95% |
|  |  |  |  |  |  | biphase - low CP | 15.9 | 89% | 81% | 83% | 93% |
|  |  |  |  |  |  | biphase - minimum CP | 13.1 | 92% | 67% | 74% | 102% |
|  |  |  |  |  |  | multiphase - no aa | 16.9 | 95% | 87% | 94% | 95% |
|  |  |  |  |  |  | multiphase - least cost with aa | 13.3 | 88% | 67% | 73% | 94% |
|  |  |  |  |  |  | multiphase - minimum CP | 12.3 | 89% | 63% | 72% | 99% |
| *Ogino et al., 2013* | *sows + fattening pigs* | *animal* | *Japan* | *North america* | *no* | conventional diets | 17.1/14 | 100% | 100% | 100% | 100% |
|  |  |  |  |  |  | low CP diets with AA | 14.5/10.8 | 94% | 95% | 72% | 101% |
